# Correlations of mutations in *kat*G, *oxy*R-*ahp*C and *inh*A genes and *in vitro *susceptibility in *Mycobacterium tuberculosis *clinical strains segregated by spoligotype families from tuberculosis prevalent countries in South America

**DOI:** 10.1186/1471-2180-9-39

**Published:** 2009-02-19

**Authors:** Elis R Dalla Costa, Marta O Ribeiro, Márcia SN Silva, Liane S Arnold, Diana C Rostirolla, Patricia I Cafrune, Roger C Espinoza, Moises Palaci, Maria A Telles, Viviana Ritacco, Philip N Suffys, Maria L Lopes, Creuza L Campelo, Silvana S Miranda, Kristin Kremer, Pedro E Almeida da Silva, Leila de Souza Fonseca, John L Ho, Afrânio L Kritski, Maria LR Rossetti

**Affiliations:** 1 State Foundation for Production and Research in Health (FEPPS), Porto Alegre, Brazil; 2 Tuberculosis Academic Program – Federal University of Rio de Janeiro, UFRJ, Brazil; 3 Luterana University of Brazil, ULBRARS, Brazil; 4 Federal University of Rio Grande do Sul, UFRGS, Brazil; 5 Blufstein Clinic Laboratory, LCB, Peru; 6 Federal University of Espírito Santo, UFES, Brazil; 7 Adolfo Lutz Institute, IAL, São Paulo, Brazil; 8 Servicio de Micobacterias, Instittuto Nacional Enfermedades Infecciosas, ANLIS, Argentina; 9 Cornell University, Ithaca, New York, USA; 10 Oswaldo Cruz Institute, Fiocruz, Rio de Janeiro, Brazil; 11 Evandro Chagas Institute, IEC, Manaus, Brazil; 12 LACEN Ceará, Brazil; 13 Mycobacteria Reference Unit (CIb-LIS), National Institute for Public Health and the Environment (RIVM), the Netherlands; 14 Federal Foundation of Rio Grande, FURG, Brazil; 15 Federal University of Minas Gerais, Brazil; 16 Centro de Desenvolvimento Científico e Tecnológico (CDCT), Fundação Estadual de Produção e Pesquisa em Saúde (FEPPS), Av Ipiranga, 5400 3° andar, CEP 90610 000, Porto Alegre, Brazil

## Abstract

**Background:**

Mutations associated with resistance to rifampin or streptomycin have been reported for W/Beijing and Latin American Mediterranean (LAM) strain families of *Mycobacterium tuberculosis*. A few studies with limited sample sizes have separately evaluated mutations in *kat*G, *ahp*C and *inh*A genes that are associated with isoniazid (INH) resistance. Increasing prevalence of INH resistance, especially in high tuberculosis (TB) prevalent countries is worsening the burden of TB control programs, since similar transmission rates are noted for INH susceptible and resistant *M. tuberculosis *strains.

**Results:**

We, therefore, conducted a comprehensive evaluation of INH resistant *M. tuberculosis *strains (n = 224) from three South American countries with high burden of drug resistant TB to characterize mutations in *kat*G, *ahp*C and *inh*A gene loci and correlate with minimal inhibitory concentrations (MIC) levels and spoligotype strain family. Mutations in *kat*G were observed in 181 (80.8%) of the isolates of which 178 (98.3%) was contributed by the *katG *S315T mutation. Additional mutations seen included *oxy*R-*ahp*C; *inh*A regulatory region and *inh*A structural gene. The S315T *kat*G mutation was significantly more likely to be associated with MIC for INH ≥2 μg/mL. The S315T *kat*G mutation was also more frequent in Haarlem family strains than LAM (n = 81) and T strain families.

**Conclusion:**

Our data suggests that genetic screening for the S315T *kat*G mutation may provide rapid information for anti-TB regimen selection, epidemiological monitoring of INH resistance and, possibly, to track transmission of INH resistant strains.

## Background

Tuberculosis (TB), a curable disease caused by *M. tuberculosis*, has never been adequately controlled in high prevalence countries because of inadequate funding of public health programs and limited access to health care caused by poverty. In the last several decades, the concurrent HIV epidemic has further accentuated the magnitude of the global TB burden. Further complicating the TB resurgence is the recent increase in the occurrence of simultaneous resistance to first line drugs, isoniazid (INH) and rifampin (RIF), that defines multidrug resistance (MDR), as well as, to second line drugs, resulting in extensive drug resistance (XDR) [1,2]. Although current control measures and short-term treatment schemes address the problem of drug resistance, knowledge on individual drug resistance profiles is needed for targeted intervention [3]. Global surveillance of *M. tuberculosis *drug resistance has been proposed to guide appropriate treatment policies [4]. Brazil and Peru are responsible for approximately 50% of the new TB cases in the Americas [5,2]. Moreover, 2,443 and 2,760 MDR-TB cases were reported respectively for Brazil from 2000 to 2006 [6] and Peru in just 2005 [7].

In the last years, molecular epidemiological approaches have shown that certain emerging *M. tuberculosis *strains, that induce more severe forms of TB, manifest higher failure/relapse than others. These features of certain isolates of *M. tuberculosis *strains, therefore, accentuate TB burden even in countries with good TB control programs, such as Vietnam [8-10]. Strains of the Beijing/W and Haarlem strain families of *M. tuberculosis *are emerging in certain global regions and are associated with drug resistance [11,12]. Importantly, specific mutations have been described in *M. tuberculosis *genes that are associated with resistance to rifampin or streptomycin and noted particularly in W/Beijing and Latin-American & Mediterranean (LAM) strain families [13].

The current view, since Middlebrook's original description, is that INH resistant strains of *M. tuberculosis *are less virulent; whether INH resistant and catalase-negative strains are indeed attenuated has been recently questioned [14]. The mechanism for INH resistance is only partly elucidated. Resistance to INH is associated with mutations in several genes that include at least *kat*G, *inh*A and *ahp*C. The *kat*G gene encodes the enzyme catalase-peroxidase that functions to convert INH, which lacks anti-mycobactericidal activity, into an active compound [15]. The *inh*A (ORF) gene encodes an enoyl acyl carrier protein reductase involved in fatty acid synthesis. These fatty acids are the target of the active derivative of INH [4]. The *inh*A promoter gene region regulates the expression of an enoyl acyl carrier protein reductase. Mutations of this region may decrease the level of protein expression. The *ahp*C gene encodes alkyl-hydroperoxide reducatse involved in cellular regulation of oxidative stress [16]; mutations in the intergenic region *oxyR-ahpC *may also reduce the level of expression. The substitution of a single nucleotide of the amino acid at position 315 of *kat*G (S→T), vary from 53% to 96% of INH resistant isolates [17,18]. Importantly, it was shown that the *kat*G S315T mutation is associated with INH resistance without diminishing the virulence or transmissibility of *M. tuberculosis *strains [3,19]. The lack of attenuation associated with the *kat*G S315T substitution and its high frequency among INH resistant clinical isolates suggests that the majority of these isolates will be virulent, and this premise was supported by a recent population-based molecular epidemiological study carried out in The Netherlands [20]. In this study, DNA fingerprinting demonstrated that, although INH resistant strains in general were less often transmitted between humans, the transmission of *kat*G S315T mutants was similar to drug susceptible strains [20,18].

There is a paucity of information regarding the frequency and types of gene mutations associated with INH resistance among *M. tuberculosis *strains from South America. Moreover, studies of mutations associated with INH resistance have been limited in the scope of the genes assessed, the number of isolates evaluated, and lacked correlation with *in vitro *INH levels determined by minimal inhibitory concentration. Thus, we conducted a comprehensive characterization of mutations in the *kat*G, *oxy*R-*ahp*C, and *inh*A genes in over 200 INH resistant *M. tuberculosis *isolates from three MDR high prevalence countries from South America, namely, Argentina, Peru and Brazil and correlated the mutational data with minimal inhibitory concentration (MIC) level for INH and strain families as determined by spoligotyping.

## Results

### Drug susceptibility testing

All isolates previously shown to be INH resistant by the proportion method were retested to determine the MIC levels. All isolates retested by MIC were INH resistant defined as ≥ 0.2 μg/mL. The majority of the isolates were resistant to ≥ 0.5 μg/mL INH.

### Mutation frequency

**Figure 1:**
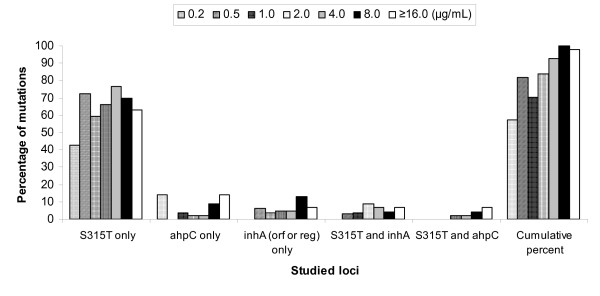
**Correlation or MIC levels and percentage of strains bearing the studied mutations in *Kat*G, *ahp*C and *inh*A gene loci**. Cumulative percent at each MIC level is derived by the number of isolates with any of the assessed mutations divided by all isolates × 100.

We next characterized mutations in *kat*G, *ahp*C and *inh*A (ORF or regulatory regions) gene loci. Among the 224 INH resistant *M. tuberculosis *isolates, the *kat*G gene was the most frequently mutated gene (80.8%; 181/224). A mutation in codon 315 of the *kat*G gene was present in 178 isolates. At this codon, the substitution from AGC to ACC leading to the amino acid change serine to threonine (S to T), seen in 166 (74.1%) isolates. In addition, a single nucleotide polymorphism (SNP) from AGC (S) to AAC (N) was seen in 9 isolates; and from AGC (S) to ACG (L) was noted for 3 isolates. In other regions of the *kat*G gene, substitution SNPs were identified at codons 258, 299 and 300 (Table 1). We also screened for mutations in *oxy*R-*ahp*C and *inh*A (ORF and regulatory) gene loci previously reported to be associated with INH resistance. Mutations were also identified including in *oxy*R-*ahp*C (8.9%, n = 20 isolates), *inh*A regulatory gene region (9.8%, n = 22 isolates), and *inh*A ORF gene region (1.3%, n = 3 isolates) (see Table 1). Figure 1 depicts correlation of MIC level with frequencies of individual mutations and cumulative mutations. As shown, 99.8% of isolates with MIC ≤ 8 μg/mL present at least one mutation. The data suggest that with increasing MIC levels, the assessed mutations could account for or is associated with an increasingly greater proportion of isolates having the quantified resistance MIC level.

**Table 1:** Mutations identified in 224 INH resistant *M. tuberculosis *isolates from South America

	Specific mutation in each loci (number of isolates with mutation)						
	*kat*G only	*Oxy*R-*ahp*C only	*inh*A (reg) only	*inh*A (ORF) only	*Kat*G and *inh*A (reg)	*Kat*G and *ahp*C	No mutation*

Brazil (176)	S315T (121) S315N (5) S315I (3) G258D*** (1)	C(-15)T (1) I20I (1)**/*** C(-39)T (3) C(-30)T (1) G(-6)A (2) G(-32)A (1)	C(-15)T (7)	G(82)R*** (1)	W300R***/C(-15)T (1) S315T/C(-15)T (8)	S315N/I20I**/*** (1) G299S/G(-9)A (1) S315T/G(-48)A (1)	17

Peru (34)	S315T (19) S315N (2)	C(-10)T (1)	C(-15)T (3)	S(94) R*** (1)	S315T/C(-15)T (1)	S315N/C(-10)A*** (1) S315T/C(-10)A*** (3) S315T/C(-15)T (1)	2

Argentina (14)	S315T (9)	C(-15)T (1) C(-10)T (1)	---	S(93)A*** (1)	S315T/C(-15)T (1)	---	1

Total 224	N = 160	N = 12	N = 10	N = 3	N = 11	N = 8	N = 20

*No mutation in studied loci.

**Silent mutation in the codon 20 of the *ahp*C gene.

***Not reported in the literature.

### Country specific mutation frequency

The proportion of *M. tuberculosis *isolates with any *kat*G mutation in the different countries was; Brazil (81.3%, n = 143), Peru (82.4%, n = 28), and Argentina (71.4%, n = 10) (p > 0.05); and the S315T *kat*G mutation was: Brazil (74.4%, n = 131), Peru (73.5%, n = 25), and Argentina (71.4%, n = 10).

### Spoligopatterns

The INH resistant *M. tuberculosis *isolates (n = 224) were spoligotyped and segregated in strain families in which 86 different spoligotype patterns were identified. We next evaluated for shared spoligotype patterns in which 158 isolates clustering by spoligotyping matched with 27 international types (SITs, which had two or more isolates in an updated SpolDB4 [21] – Table 2). Other 30 isolates matched 30 individual SITs, reported as orphans by SpolDB4, Table 2. A third group of isolates (n = 36 [16.0% of the tested isolates] segregated into 29 newly identified spoligotype patterns (not reported by SpolDB4). The strain families that could be grouped by SpolDB4 included: LAM (46.4%, n = 104), Haarlem (16.0%, n = 36), T (14.3%, n = 32), X (6.2%, n = 14), S (4.5%, n = 10), U (4.9%, 11), W/Beijing (1.8%, n = 4), MANU2 (0.4%, n = 1). Twelve (4.8%) isolates had an unclassified spoligopattern. Five isolates were included as Haarlem because of their spoligotype signature but did not match any of the patterns in SpolDB4 [21].

**Table 2:** Frequency of 27 shared spoligotypes (SITs) according to Brudey *et al*. [21] identified in 158 INH resistant *M. tuberculosis *strains isolated from South America.

SIT	Octal	Strains in this n		Strains in n		Lineag
1	0000000000037	3	1.3	5610	13.2	Beijing
47	7777777740207	6	2.6	1021	2.4	Haarlem
602	7777777700007	2	0.9	48	0.1	U
50	7777777777207	19	8.5	2128	5.0	Haarlem
49	7777777777207	3	1.3	115	0.3	Haarlem3
20	6777776077607	9	4.0	588	1.4	LAM
17	6777376077607	6	2.4	473	1.1	LAM
33	7761776077607	8	3.6	770	1.8	LAM
4	0000000077607	3	1.3	220	0.5	LAM3/S
211	5761776077607	2	0.9	63	0.1	LAM
828	3777776077607	3	1.3	20	0.0	LAM
93	7777376077607	10	4.5	267	0.6	LAM
64	7777776075607	9	4.0	157	0.4	LAM
435	7637776077607	3	1.3	4	0.0	LAM
177	3777776077607	3	1.3	50	0.1	LAM
388	7377776077607	2	0.9	15	0.0	LAM
42	7777776077607	22	9.9	1926	4.5	LAM
1938	7763777777607	7	3.1	3	0.0	S
53	7777777777607	17	7.6	3738	8.8	T1
397	7777776000007	2	0.9	13	0.0	U
402	7777776000000	3	1.3	14	0.0	U
1241	7777776077007	3	1.3	28	0.0	U
119	7777767777607	2	0.9	659	1.8	X1
137	7777767777606	3	1.3	720	2.0	X2
92	7000767777607	3	1.3	328	0.8	X3
91	7000367777607	2	0.9	143	0.4	X3
60	7777776077607	3	1.3	83	0.2	LAM

### Association between MIC levels, characterized mutations and spoligotype strain families

Higher level INH resistance (≥2 μg/mL) was significantly associated with the S315T *kat*G mutation, as shown by a greater odds ratio of 1.97 (Table 3). Of note, in isolates with MIC ≥16 μg/mL (83.0%, n = 38) a mutation was found one or more of the studied genes. We next evaluated for potential the relationship between MIC levels and mutations and strain families. The S315T *kat*G mutation was found in LAM isolates (77.9%, n = 81), Haarlem isolates (94.4%, n = 34), and in T isolates (68.7%, n = 22). Of the Beijing strains (n = 4), 3 presented with the S315T *kat*G mutation. We noted a statistical association between Haarlem strain family with the S315T *kat*G mutation (p = 0.01) (Table 3). When the specific S315T *kat*G mutation was considered, the Haarlem genotype occurred more frequently among those *M. tuberculosis *strains with MIC ≥2 μg/mL (p = 0.02). The most frequent Haarlem spoligotype pattern was the shared international type (SIT) 50, which was found in 19 (52.7%) isolates and only one of these did not possess the S315T *kat*G mutation. LAM strain family showed the highest frequency (46.2%, n = 104) among the 224 isolates which were distributed among 20 different SITs according to spolDB4 (Table 4). The LAM9 lineage was the most frequent LAM lineage (29.8%) identified in all three countries studied. In contrast to Haarlem, the LAM strain family was not associated with the S315T *kat*G mutation (p = 0.58) nor with higher MIC values (p = 0.79) (Table 3). Among T family strains, 6 (18.8%) isolates were related to sub-clades T2, T3, T4 and T5; 22 (68.7%) isolates had the S315T mutation.

**Table 3:** 315 mutation and its correlation with MIC and spoligotype families distribution in INH resistant *M. tuberculosis *isolates

	315 m	315 w	P value	OR (CI 95%)
MIC ≥2.0 μg/ml				
Yes	127	26	0.05	1.92 (0.93–3.93)
No	51	20		
Haarlem				
Yes	34	2	0.01	5.19 (1.15–3.25)
No	144	44		
LAM				
Yes	81	23	0.58	0.84 (0.42–1.68)
No	97	23		
T				
Yes	22	10	0.45	0.73 (0.30–1.80)
No	144	48		

315 m: mutated; 315 w: wild type

**Table 4:** Frequency of 57 shared spoligotypes (SITs) according to Brudey *et al*. [21], identified in 188 *M. tuberculosis *strains isolated with S315T mutation, from South America.

Lineage	SIT	Number of isolates	Number of DRE-PCR patterns	Number of isolates in cluster (%)
Beijing	1	3	2	2 (66.7)

Haarlem1	47	6	6	0

Haarlem3	50	19	16	6 (31.5)

Haarlem3	49	3	2	2 (66.7)

LAM1	20	9	4	7 (77.8)

LAM2	17	6	3	3 (50.0)

LAM3	33	8	4	4 (50.0)

LAM3/S convergent	4	3	2	2 (66.7)

LAM3	211	2	1	2 (100)

LAM4	828	3	3	0

LAM5	93	10	6	4 (40.0)

LAM6	64	9	6	2 (22.2)

LAM9	435	3	1	3 (100)

LAM9	177	3	3	0

LAM9	388	2	1	2 (100)

LAM9	42	22	12	14 (63.6)

S	1938	7	5	4 (57.0)

T1	53	17	11	4 (23.5)

U	397	3	2	2 (66.7)

U	402	2	2	0

U	1241	3	3	0

X1	119	3	3	0

X2	137	2	2	0

X3	92	3	2	2 (66.7)

X3	91	3	3	0

LAM4	60	2	2	0

U	602	2	2	0

T5-MAD2	58	1	Not done	Unique

LAM6	95	1	Not done	Unique

U (LAM3?)	106	1	Not done	Unique

T5	68	1	Not done	Unique

T3	157	1	Not done	Unique

T1	159	1	Not done	Unique

Haarlem3	207	1	Not done	Unique

T1	253	1	Not done	Unique

Beijing-Like	269	1	Not done	Unique

T1	353	1	Not done	Unique

T1	453	1	Not done	Unique

Haarlem3	631	1	Not done	Unique

S	707	1	Not done	Unique

S	827	1	Not done	Unique

LAM5-LAM6	867	1	Not done	Unique

X2	1341	1	Not done	Unique

T2	1355	1	Not done	Unique

LAM9	1535	1	Not done	Unique

LAM2	1691	1	Not done	Unique

LAM1	389	1	Not done	Unique

T1	276	1	Not done	Unique

Haarlem3	294	1	Not done	Unique

MANU2	1094	1	Not done	Unique

LAM5	176	1	Not done	Unique

T3	1655	1	Not done	Unique

LAM3	130	1	Not done	Unique

T4-CE1 ancestor?	65	1	Not done	Unique

Haarlem3-LAM9	335	1	Not done	Unique

T1	222	1	Not done	Unique

LAM3	1354	1	Not done	Unique

### Frequency of INH resistance associated mutation in spoligotype strain families

To evaluate for genetic correlation of strains with the same spoligopatterns, DRE-PCR was performed on isolates presenting the same INH conferring mutation and the same spoligotype. DRE-PCR has previously been used to genetically classify strains with the same spoligotyped as being genetically related (or clustered isolates). The most frequently observed spoligotype patterns among isolates with the S315T *kat*G mutation were SIT 42 (LAM9, 22 isolates) and SIT 50 (Haarlem3, 19 isolates). Among the isolates that had a SIT 42 spoligotype pattern and a S315T *kat*G mutation, 12 different DRE-patterns were identified, presenting 14 (63.6%) isolates in four different clusters and 8 unique isolates. The isolates with a SIT 50 spoligotype showed 16 different DRE-patterns, presenting 6 (31.5%) isolates in three different clusters and 13 unique isolates (Table 4).

In total, 62 (27.6%) of S315T *kat*G mutated isolates appeared distributed in 29 clusters, most of them with just two isolates per cluster. Of the INH resistant strains that did not have the S315T *kat*G mutation, 19 (27.9%) were in clusters. The proportion of clustering was higher among LAM lineage *M. tuberculosis *isolates (40.7%; 33/81) carrying the S315T *kat*G mutation than in LAM isolates without the S315T *kat*G mutation (26%; 7/23). A higher proportion of clustering in which the S315T *kat*G mutation was also noted for the few W/Beijing strains (50% (2/4). In contrast, the proportion of clustering in S315T *kat*G mutated was lower for Haarlem isolates (23.5%, 8 of 34), T (18%, 4 of 22).

## Discussion

Identification of markers for rapid determination of TB drug resistance is needed to combat the increasing prevalence of MDR TB. Mutations in select genes of *M. tuberculosis *have been used as correlates for anti-TB drug resistance. Prior reports have evaluated in a limited setting one or more of the gene loci evaluated by this report including, *kat*G, *ahp*C, regulatory region of *inh*A, and the ORF region of *inh*A. However, none of these studies have comprehensively catalogued mutations in all of these loci in a single study and testing large numbers of clinical samples from TB prevalent regions such as, South America, nor have they correlated the identified mutations with INH MIC levels.

In this study, each clinical isolate was characterized for mutations not only in *kat*G gene, but also in *ahp*C, regulatory region of *inh*A, and ORF region of *inh*A. Frequencies of *kat*G mutation among INH resistant *M. tuberculosis *isolates in three South American countries was: Brazil (81.3%), Peru (82.4%) and, Argentina (71.4%). Our study does not aim to provide a profile of the involved sites, but to characterize mutations from the available strains during the period. The frequency for the *kat*G S315T mutation in INH resistant *M. tuberculosis *isolates was comparable to the previously reported rate for patients diagnosed in Kuwait, Brazil and The Netherlands (65% and 55%, respectively) but was lower than described in Russia (95%) [13,20,22,23].

In this study, we also correlated MIC levels with the *kat*G S315T mutation in INH resistant *M. tuberculosis *isolates. We demonstrated that 83.0% (n = 127) of the INH resistant strains with the *kat*G S315T mutation possessed a MIC for INH ≥2 μg/mL (p = 0.05). These data are in accordance with The Netherlands report, where 95% of the INH strains with this mutation had a MIC for INH of > 2 μg/L (20). The mutation AGC to ACC at codon 315 tended also to be associated with MIC ≥2 μg/mL (p = 0.06; OR = 1.79 [confidence interval (CI): 0.92–3.49]). Part of the success of the *kat*G S315T mutated isolates in the community is probably because the catalase-peroxidase enzyme is still active in these mutants; indeed, 30% to 40% of the initial catalase activity remains when this mutation is introduced into the *kat*G gene by site-directed mutagenesis [19,24].

Mutations in coding or regulatory regions of other genes such as the *oxy*R-*ahp*C region have also been associated with INH resistance, but occur less frequently [1]. Mutations of the *oxy*R-*ahp*C region have been described in 4.8% to 24.2% of INH resistant *M. tuberculosis *isolates [25-27,15]. Usually, higher levels of INH resistance and/or loss of catalase activity are associated with mutations in *inh*A and *ahp*C genes [28,29]. In the present study, few isolates had mutations in more than one gene. Eight isolates (3.6%) had mutations in both *kat*G and *oxy*R-*ahp*C; 5 from Peru and 3 from Brazil (Table 1). Of note, *M. tuberculosis *isolates with the *kat*G S315T mutation and *inh*A or *ahp*C, or *inh*A and *ahp*C genes tended to occur more frequently in isolates with a MIC for INH of ≥2 μg/mL, appearing in 22 isolates (p = 0.06; OR 0.95–4.8). After the *kat*G gene, the *inh*A promoter gene was the second most frequently mutated gene, with mutation in 10% of the *M. tuberculosis *isolates. This frequency is in accordance to others, varying from 10% to 34.2%, described elsewhere [30,31]. All mutations occurred in the regulatory region of the *mab*A-*inh*A operon with a C to T change at position -15, reported to be associated with INH resistance [32,28]. Similarly as has been previously described by others, few mutations were identified in the *inhA *ORF [4,23].

Frequencies of *M. tuberculosis *lineage found in our study were in range with frequencies described in recently published population-based studies performed in other South American countries [33,34]. LAM family was the most frequent lineage found by this study, occurring among 46.4% of the INH resistant *M. tuberculosis *isolates in our South American study population. This proportion is virtually identical to that found among INH resistant *M. tuberculosis *isolates from Russia [13]. The Haarlem family was the second most frequent family, with a similar proportion of isolates belonging to the Haarlem family as reported in in Russia (10%) [12]. A high frequency of the *kat*G S315T mutation in INH resistant *M. tuberculosis *isolates of the Haarlem strain family was also described in South Africa [12] and Tunisia [35]. As with the W/Beijing family, the Haarlem family is widespread [36], and has mutations within putative mutator genes [37,38]. Mutation in such genes may afford these strains a higher adaptability to hostile environments, following challenge by anti-TB drugs or engulfment within macrophages [38]. The Haarlem family appears to favor the emergence of MDR-TB strains, and was associated with outbreaks in Argentina [39], the Czech Republic [40] and Tunisia [35]. W/Beijing family strains, which are often associated with drug resistance, although prevalent in many regions of the world, are mostly localized in Asia and Eastern European countries [11,8,41,42], and, at present, uncommon in Latin American countries [33,34,43,44], which was confirmed by this study (only five W/Beijing isolates were identified). The T family occurred in 14.3% of our INH resistant *M. tuberculosis *isolates, which is similar to the proportion reported in Paraguay (8.6%) and in Venezuela (13%) [22,34]. As a descriptive study on selected *M. tuberculosis *isolates that were provided by the reference TB laboratories from different regions in Latin America, its limitation rely on the lack of generazibility. The available *M. tuberculosis *isolates included in the project have no aiming to be a representative from each country on the mutations profiles of INH resistant *M. tuberculosis *isolates. The second phase of this study is underway: the evaluation of same techniques using randomly INH sensitive and INH resistant *M. tuberculosis *isolates isolated at National Drug Resistant Surveillance carried out in those countries in the last years.

Even though the application of DOTS has stabilized the prevalence of TB or has led to decline in some countries, drug-resistant TB is rapidly emerging in a significant number of areas in the world [2]. Under standard treatment regimens it is often not possible to identify primary drug-resistant cases and these regimens are therefore unsuitable for the control of drug-resistant strains. TB control thus relies on improving current TB diagnosis and early detection of drug-resistant TB, preferably using rapid and accurate screening tools other than the sole reliance on AFB smear and culture identification and susceptibility testing.

## Conclusion

The present data indicate that screening for the *kat*G S315T mutation may be useful in South America for an early detection of INH resistance and, hence, provide rapid information for selection of appropriate anti-TB therapy. This information may also be used as a marker to evaluate the transmissibility of INH resistant TB in the community. Our study also demonstrated an association between a high MIC and *kat*G S315T mutation, as well as an association between the *kat*G S315T mutation, and Haarlem strain family that may in part explain the successful spread of Haarlem strains in South America.

## Methods

The present experimental research that is reported in the manuscript has been performed with the approval of an appropriate ethics committee and carried out within an ethical framework.

### Mycobacterial strains

The *M. tuberculosis *isolates and respective data of INH susceptibility tests were kindly provided by the National Health Institute in Peru (n = 34), the Malbran Institute (n = 14) in Argentina and from seven Brazilian Institutes: Ceará State (CE) Central Laboratory (n = 25), Central Laboratory of Rio Grande do Sul State (RS, n = 24); Federal University of Rio de Janeiro (RJ, n = 32); Federal University of Espírito Santo (ES, n = 31); Adolfo Lutz Institute of Paulo State (SP, n = 23); Federal University of Minas Gerais (MG, n = 27); Evandro Chagas Institute, Pará (PA) (n = 14). These were the total number of strains provided by each site included in this study. All strains were collected from September 2003 to December 2004 and were identified to the species level by analysis of morphologic and biochemical characteristics [45]. Reference strain *M. tuberculosis *H37Rv ATCC 27294 was used as a control INH susceptible strain. The strains and the reference strain were tested for susceptibility by each site using the proportion method on Lowenstein-Jensen (LJ) medium [46] in the absence and presence of 0.2 μg/ml for INH or no INH.

### Minimum inhibitory concentration (MIC) determination

The test was performed as described by Palomino *et al*, 2002 [47]. The INH (Sigma, St. Louis, MO, USA) stock solution was prepared at concentration of 10 mg/mL in sterile distilled water. Serial two-fold dilutions of INH in 100 μL of Middlebrook 7H9 broth medium (Difco, Detroit, MI, USA) containing glycerol enriched with 10% oleic acid-albumin-dextrose-catalase (OADC) and Bacto Casitone (Difco) were prepared directly in 96-well flat-bottom microplates (Corning Costar, Cambridge, MA, USA) at final INH concentrations from 16 to 0.2 μg/mL (200 μL total volume). The inoculum was prepared from fresh LJ medium in Middlebrook 7H9 broth medium adjusted to a McFarland symbol.1 and then further diluted 1:20. A 100 μL aliquot of this dilution was added into each well. The microplates were covered, sealed in plastic bags, and incubated at 37°C in the normal atmosphere. After 7 days of incubation, 30 μL of resazurin solution was added to each well, incubated overnight at 37°C, and assessed for color development. Resazurin sodium salt powder (Acros Organic N.V.) prepared at 0.01% (wt/vol) in distilled water was used as a general indicator of cellular growth and viability. A change from blue to pink indicates reduction of resazurin and therefore bacterial growth. The MIC was defined as the lowest drug concentration that presented no color change. The cut off value for resistance was ≥ 0.2 μg/mL according Palomino *et al*, 2002 [32]. Growth controls containing no INH and sterility controls without *M. tuberculosis *were included in each MIC testing.

### Nucleic acid extraction

Chromosomal DNA was extracted from cultures on Löwenstein-Jensen medium, using the CTAB method as described by van Embden *et al*., 1993 [48].

### Sequence analysis

The genes were amplified with the following primers (*Kat*G 1. – 5' CAT GAA CGA CGT CGA AAC AG 3', *Kat*G 2. – 5' CGA GGA AAC TGT TGT CCC AT 3'; *ahp*C 1. – 5' GCC TGG GTG TTC GTC ACT GGT 3', *ahp*C 2. – 5' CGC AAC GTC GAC TGG CTC ATA 3'; *inh*A (ORF) 1. – 5' GAA CTC GAC GTG CAA AAC 3', *inh*A (ORF) 2. – 5' CAT CGA AGC ATA CGA ATA 3'; *inh*A (reg) 1. – CCTCGCTGCCCAGAAAGGGA, *inh*A (reg) 2. – ATCCCCCGGTTTCCTCCGGT), yielding fragments of 232 bp, 359 bp, 206 bp and 248 bp, respectively. Amplifications were carried out in a thermocycler Mini-Cycler-Hot Bonnet PTC-100 (MJ Research, INC, EUA) as follows: 94°C for 2 min, 55°C for 1 min, and 72°C for 2 min, for 30 cycles. Amplification products were analyzed by electrophoresis in 1.5% agarose gels, purified with MicroSpin S-300 HR Columns (Amersham Biosciences, Piscataway, NJ, USA) and sequenced by using the Big Dye Terminator Cycle Sequencing Kit with AmpliTaq DNA polymerase (Applied Biosystems, Foster City, CA, USA) in the ABI Prism 3100 DNA Sequencer (Applied Biosystems).

### Spoligotyping

Spoligotyping was performed as described by Kamerbeek *et al *[49,21]. To determine the spoligotype family, patterns were compared to those in the international database of spoligo patterns (SpolDB4). The double repetitive element (DRE) PCR was performed in accordance to Friedman, 1995 [50]. The term 'cluster' was used for two or more *M. tuberculosis *isolates with identical spoligotype and DRE-PCR patterns.

### Statistical analysis

Data were analyzed using Epi Info (version 6.03, CDC, Atlanta, GA, US; public domain). Categorical variables were compared by the Fisher exact or chi-squared test. A confidence interval (CI) of 95% was used in all odds ratio (OR) calculations.

## Authors' contributions

ERDC: carried out the molecular genetic studies, participated in genotyping studies, analyzed the data and wrote the manuscript. MSNS: contributed to drafting the manuscript and provided suggestions during manuscript preparation. LSA: participated in the molecular genetic studies. DCR: participated in genotyping studies. PIC: carried out the genotyping studies. MAT, MP: carried out mycobacteriological diagnostics, isolation, identification and drug susceptibility testing of clinical isolates, and provided critical comments for the manuscript. VR, KK, PEAS: provided critical comments for the manuscript. PNS: participated in the design of the study and provided critical comments for the manuscript. MLL, CLC, SSM, RCE, MOR: carried out mycobacteriological diagnostics, isolation, identification and drug susceptibility testing of clinical isolates. LSF, JLH: participated in the design of the study and provided critical comments for the manuscript. ALK, MLRR: conceived the study and the methodology, coordinated the investigation and wrote the manuscript. All authors read and approved the final manuscript.
